# Ensemble of ROI-based convolutional neural network classifiers for staging the Alzheimer disease spectrum from magnetic resonance imaging

**DOI:** 10.1371/journal.pone.0242712

**Published:** 2020-12-08

**Authors:** Samsuddin Ahmed, Byeong C. Kim, Kun Ho Lee, Ho Yub Jung, for the Alzheimer’s Disease Neuroimaging Initiative

**Affiliations:** 1 Department of Computer Engineering, Chosun University, Gwangju, South Korea; 2 Gwangju Alzheimer’s disease and Related Dementias Cohort Research Center, Chosun University, Gwangju, Korea; 3 Department of Biomedical Science, Chosun University, Gwangju, South Korea; 4 Department of Neurology, Chonnam National University Medical School, Gwangju, South Korea; 5 Korea Brain Research Institute, Daegu, Korea; Nathan S Kline Institute, UNITED STATES

## Abstract

Patches from three orthogonal views of selected cerebral regions can be utilized to learn convolutional neural network (CNN) models for staging the Alzheimer disease (AD) spectrum including preclinical AD, mild cognitive impairment due to AD, and dementia due to AD and normal controls. Hippocampi, amygdalae and insulae were selected from the volumetric analysis of structured magnetic resonance images (MRIs). Three-view patches (TVPs) from these regions were fed to the CNN for training. MRIs were classified with the SoftMax-normalized scores of individual model predictions on TVPs. The significance of each region of interest (ROI) for staging the AD spectrum was evaluated and reported. The results of the ensemble classifier are compared with state-of-the-art methods using the same evaluation metrics. Patch-based ROI ensembles provide comparable diagnostic performance for AD staging. In this work, TVP-based ROI analysis using a CNN provides informative landmarks in cerebral MRIs and may have significance in clinical studies and computer-aided diagnosis system design.

## Introduction

The National Institute on Aging and Alzheimer’s Association (NIA-AA) defines three stages of AD on the basis of pathobiology and clinical symptoms [1]. The stages are a) preclinical AD or asymptomatic predementia (aAD) b) MCI due to AD (mAD) and c) AD dementia (ADD). The brain contains beta-amyloid outside the neuronal cells and tau tangles inside the neurons in different phases of AD [2, 3]. Unlike mAD and ADD, the aAD stage is not associated with cognitive symptoms.

In addition to clinical evaluation and psychological tests, artificial intelligence (AI)-based computer-aided diagnosis (CAD) methods for staging AD from structured magnetic resonance imaging (sMRI) have been developed [4–17]. Conventional AI techniques require domain expertise and careful engineering for feature extraction [18]. In contrast, deep learning (DL)-based methods are well recognized for their representation learning capability [18]. As a result, recent trends in AD diagnosis include the use of DL-based approaches. DL-based [7, 11, 12, 19–21] studies consider multimodal information for classifying AD and mAD from NC. The studies [7, 22, 23] use 3D patches from the whole brain to train and test a CNN model. There are [24] studies that also discuss 2*D* + *ϵ* methods that incorporate multiview patches of brain sMRI for diagnosing AD.

However, DL-based methods require a sufficient quantity of training data for generalization, specifically for expressing highly complex problems such as AD staging. Due to difficulty in data acquisition and quality annotation, the data scarcity problem is considered one of the main limiting factors of AD classification [25]. Medical imaging studies [26–28] have attempted to avoid the data scarcity issue by sufficient patch generation, which has also been practiced in AD research [14, 29]. The patch generation from any voxel location of the brain may not provide the discerning information. However, clinicians have suggested that, in its early stage, AD causes structural atrophy to some regions. Some visual features of these regions are more important than others to understand the AD spectrum. Generating patches from these regions benefits solving the data scarcity problem and provides robust performance.

To the best of our knowledge, we are the first to propose three-view patch (TVP)-based ROI ensembles for AD spectrum staging using a CNN. In this effort, rather than using multimodal information, we have performed our experiment on sMRI. It is worth mentioning that sMRI provides detail information about the anatomical structures and morphology of brain tissues such as white matter, gray matter and cerebrospinal fluid (CSF) [30]. Therefore, it is possible to learn discernible features related to abnormal tissue atrophy and other biomarkers [31] that are sensitive to AD. In addition to providing significant biomarkers, sMRI is cost-effective and has no major side effects experienced by the participants. Some studies showed significant improvements in early diagnosis of AD by examining biological markers in sMRI [31–33]. Therefore, developing automatic image analysis methods based on sMRI may provide significant insights about ROIs.

Our objective here is to focus on selective ROIs for staging AD into NIA-AA specified phases. Statistical analysis, i.e., p-values from the permutation test, on volumetric measures was performed to select significant ROIs. Our primary aim is to use the most affected regions of the cerebral sMRI to achieve state-of-the-art results by deploying a TVP-based CNN (TVPCNN). We have exploited the Gwangju Alzheimer and Related Dementia (GARD) cohort data set and deployed lightweight CNNs for learning ROI-based binary classifiers. The classifiers were ensembled for staging an sMRI scan. We have performed a permutation test to select 3 pairs of ROIs from 101 different ROIs in the data set. TVPs of size 32 were generated from the selected ROIs for training and testing.

Our study demonstrated that hippocampi, amygdalae and insulae provide significant features for mAD and ADD. We have observed that the hippocampi are the most affected regions, followed by amygdalae and insulae. We also observed that the proposed TVPCNN could not find representative features to diagnose aAD from these ROIs in the sMRI modality at the prescribed settings.

In section 2, we briefly describe our data set including demographic characteristics and the preprocessing protocol. Section 3 presents the methodology of the study. ROI selection and model design are discussed here. The experimental setup, presented in section 4, includes ground truth preparation, data set separation and hyperparameter settings for training and validation of the models. The results and findings of each model are described in section 5. The overall discussion and comparison with state-of-the-art methods are presented in section 6. Section 7 concludes the article.

## Data set

In this study, we have exploited Gwangju Alzheimer Research Data (GARD) [34–36] and Alzheimer Neuroimaging Initiative Data. ADNI was exploited for comparison with stat-of-the-art methods while extensive analysis was done for GARD database.

### GARD dataset

GARD is a portion (326 baseline scans) of a large cohort prepared at the National Research Center for Dementia (NRCD), Chosun University, Gwangju, South Korea. The sMRI scans were acquired from the registered subjects at the NRCD during the time period of 2014 to March 2018. The subject selection, MRI acquisition and exclusion criteria are mentioned in [34–36].

#### Subjects

The clinical labels of the scans are cognitive normal (CN), amnestic mild cognitive impairment (aMCI), nonamnestic mild cognitive impairment (naMCI) and Alzheimer disease (AD). There are 206 CN scans, of which 108 subjects are female and the rest are male. Considering the presence of beta-amyloid on the positron emission tomography (PET) scans of these subjects, these 206 scans were divided into two NIA-AA defined categories, namely, aAD (35) and NC (171). The aMCI class includes 30 scans (female: male = 10: 20), and the naMCI class includes 9 scans (female: male = 4: 5). These two classes are merged into the mAD class for analysis. The AD class is renamed as the ADD class and includes 81 scans with 42 females and 39 males.

The ages of the subjects vary from 49 years to 87 years, and more than 88% subjects are older than 65 years. The education level of the participants varies from illiterate to highly educated (score 0 to 22). Table 1 briefly summarizes the data set under investigation.

**Table 1:** Selected number of MRI from different classes for training and testing.

Clinical Diagnosis	No. of Scans	Beta-Amyloid	Clinical Dementia Rating (CDR)	Education	Age	New Label (No. of scans(M/F))
Cognitive Normal (CN)	260	-	0	16(5.54)	71.66(5.43)	NC (171)
		+	0	7.88(6.30)	72.72(4.82)	aAD (35)
Amnestic Mild Cognitive Impairment (aMCI)	30	+	0.5 to 1	8.3(4.79)	73.21(8.24)	mAD (39)
Nonamnestic Mild Cognitive Impairment (naMCI)	9	+	0.5 to 1	8.3(4.79)	73.0(2.91)	
Alzheimer Disease (AD)	81	+	1 to 3	7.34(4.86)	71.96(7.08)	ADD (81)

#### Preprocessing

The sMRI scans were processed using the FreeSurfer software (FSS) version 5.3.0 [37] with an automated reconstruction protocol described in [38–40]. Pure volume (P), percentile of intracranial volume (V) and cortical thickness (T) of 101 ROIs for each scan were assessed using the measurement techniques described in [34–36]. The test-retest reproducibility of each quantitative measure was assessed. We determined the reliability of the data using Cronbach’s alpha. For Cronbach’s alpha, *α* = 0.80219 indicates acceptable reliability of the data.

#### Data availability

GARD is currently not publicly available for distribution.

### ADNI dataset

The ADNI was launched in 2003 as a public-private partnership. The primary goal of ADNI has been to test whether MRI, positron emission tomography (PET), other biological markers, and clinical and neuropsychological assessment can be combined to measure the progression of mild cognitive impairment and early Alzheimer’s disease (AD).

#### Subjects

From ADNI dataset we have selected 60 subjects aged between 65 and 96. The selected participants met the criteria defined in the ADNI protocol. There are 351 scans of these 60 subjects. There are 22 NC subjects of which 12 are males and 10 are females. The age are ranged between 62 to 90 years with mean 74.3 years and standard deviation of 3.6 years. The mini-mental state estimation (MMSE) score is 29.2 with standard deviation of 1.0. The number of MCI subjects are 18 who had not converted to AD within 18 months among which 11 are males and 7 are females with average age 70.4 with standard deviation of 3.2 years. The MMSE score is 27.2. Number of AD subjects is 20 among which 9 are males and 11 are females with average age 74.0 and standard deviotion of 5.3 years. The MMSE score is 23.2 with standard deviation 2.0.

#### Preprocessing

The raw data were provided in NII format in the ADNI database. The scans were processed using the FreeSurfer software (FSS) version 5.3.0 [37] The ROI locations were generated by DKT protocol described in [41].

#### Data availability

ADNI data is available at http://adni.loni.usc.edu/.

## Methods

The study protocol was approved by the Institutional Review Board of Chosun University Hospital, Korea (CHOSUN 2013-12-018-070). All volunteers or authorized guardians for cognitively impaired individuals gave written informed consent before participation.

**Fig 1:**
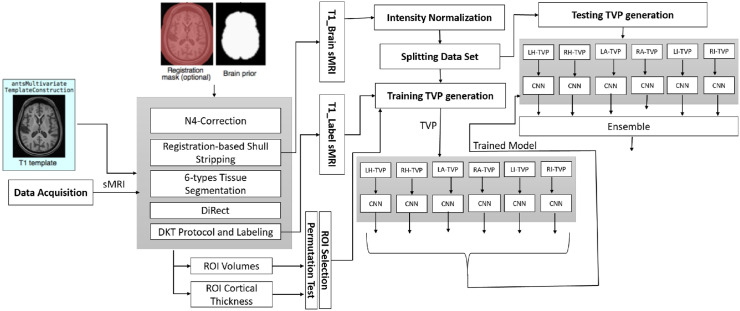
Pipeline for Alzheimer disease staging from structured magnetic resonance imaging (sMRI). TVP means three-view patch; LH, RH, LI, RI, LA and RA are the acronyms for left hippocampus, right hippocampus, left insula, right insula, left amygdala and right amygdala, respectively.

In the proposed approach, we first performed statistical analysis on the T and V measures of the studied data set to identify the most significant ROIs. Second, TVPs from axial, sagittal and coronal slices each of size 32 × 32 were produced from these ROIs for training the CNN classifiers. Each ROI-based model is evaluated to find the contributing score in the final classification. Ultimately, the trained binary classifiers are ensembled. Fig 1 briefly illustrates the pipeline, and the following subsections elaborate the concepts.

### Region of interest selection

From 101 regions labeled in GARD segmented data, we have selected 6 regions (3-pairs) based on the distinguishing capacity of the VT (percentile of intracranial volume, thickness) measures of the regions. The distinguishing capacity was measured by p-values obtained from the permutation test [42] on the given data. The p-value tests the null hypothesis that the VT measures of a specific region from two different groups (AD vs. NC) of sMRIs are identical. We have found the left hippocampus (LH), right hippocampus (RH), left amygdala (LA), right amygdala (RA), left insula (LI) and right insula (RI) to be the most significant regions. As gray matter and cortical thickness are measured on the whole brain, these two biomarkers are not studied here. The V measures of these regions provided the lowest p-values in the permutation test. The p-values for V measures are LH = 7.50e-23, RH = 3.25e-17, LI = 6.74e-11, RA = 4.63e-9, LA = 1.33e-8. The p-values of these regions for T measures are LH = 0.00149, RH = 3.74e-6, LI = 1.48e-11, RA = 1.73e-8, LA = 1.22e-12.

### Patch generation

Let any MRI, *I* = {*v* = (*v*_*x*_, *v*_*y*_, *v*_*z*_) | *v is a voxel location*}. The three principle planes (axial, sagittal and coronal) at the voxel are defined by

$$\begin{array}{c} axial:z=v_{z}\\ coronal:y=v_{y}\\ sagittal:x=v_{x} \end{array}$$ (1)

The corresponding patch of size *α* × *β* is defined by:

$$\begin{array}{c} axial\_patch:\{(x,y)|(x,y)\;is\;a\;pixel\;on\;axial\;plane\;satisfying\;\\ \;v_{x}-\frac{\alpha }{2}\leq x\leq v_{x}+\frac{\alpha }{2}\;\;and\;\;v_{y}-\frac{\beta }{2}\leq y\leq v_{y}+\frac{\beta }{2}\} \end{array}$$ (2)

$$\begin{array}{c} coronal\_patch:\{(x,z)|(x,z)\;is\;a\;pixel\;on\;coronal\;plane\;satisfying\;\\ \;v_{x}-\frac{\alpha }{2}\leq x\leq v_{x}+\frac{\alpha }{2}\;\;and\;\;v_{z}-\frac{\beta }{2}\leq z\leq v_{z}+\frac{\beta }{2}\} \end{array}$$ (3)

$$\begin{array}{c} sagittal\_patch:\{(y,z)|(y,z)\;is\;a\;pixel\;on\;sagittal\;plane\;satisfying\;\\ \;v_{y}-\frac{\alpha }{2}\leq y\leq v_{y}+\frac{\alpha }{2}\;\;and\;\;v_{z}-\frac{\beta }{2}\leq z\leq v_{z}+\frac{\beta }{2}\} \end{array}$$ (4)

The TVP at *v*(*v*_*x*_, *v*_*y*_, *v*_*z*_) is then formed by

$$\begin{array}{c} TVP_{v}\;=\;\left[axial\_patch\;coronal\_patch\;sagittal\_patch\right] \end{array}$$ (5)

By using Eq 5, we have an *α* × *β* × 3 patch. Here, *α* = *β* = 32. The class label of *I* is the class label of *TVP*_*v*_.

### Patch-based classification

The main problem of AD diagnosis is the scarcity of data. We have a limited number of samples from each class. On the other hand, it is well known that CNNs are highly susceptible to the sample size. The classification accuracy of CNNs is subject to the discriminating features among the available classes [43]. The availability of discriminating features of a class depends on the number of samples from the class. In contrast, the scarcity of data may lead to an overfitted model.

Recently, patch-based techniques have been widely used in medical imaging to solve data scarcity issues. Their application areas span from segmentation, noise removal, super-resolution, anomaly detection, disease diagnosis to image synthesis and many more [14, 27, 44–47]. In this study, we have used TVPs from the ROI. Producing TVPs facilitates acquiring sufficient training data. In addition to solving the data scarcity problem, TVP-based processing assists us to design a lightweight CNN model.

**Algorithm 1**: Algorithm for Training Data Preparation

**Data**: I = {*I*_1_, *I*_2_, *I*_3_, …*I*_*n*_} a set of sMRI scans;

Labelled sMRI, $\text{S}={⋃}_{i=1}^{n}R_{i}$ such that *R*_*i*_ is a region of the brain and *R*_*i*_ ∩ *R*_*j*_ = ∅ for any two regions *R*_*i*_ and *R*_*j*_; RList = {Left Hippocampus: 17, Left Amygdala: 18, Right Hippocampus: 53, Right Hippocampus: 54, Left Insula: 1035, Right Insula: 2035}

**Result**: D = {*x*, *y*, *l*} where *x* is TVP, y is label and l is ROIlabel

1 *D* = {}

2 **for** *each scan i* ∈ *I* **do**

3  y = label(i)

4  **for** *each voxel* (*x*, *y*, *z*) ∈ *i* **do**

5   *l* = *S*(*x*, *y*, *z*)

6   **if** *lϵRList* **then**

7    *TVP* = [*axial*_*patch*, *coronal*_*patch*, *sagittal*_*patch*]       /* Determined by Eq 5 */

8    *D* = *append*(*D*, [*TVP*, *y*, *l*])

9  **end**

10 **end**

11 return D

Our TVP-based CNN consists of convolution and pooling layers. There are three convolution layers and two fully connected layers in the model. Each convolution layer and fully connected layer are preceded by batch normalization, excluding the first and last layers. The reason for not using batch normalization before the first layer is that the inputs are normalized previously so that the mean intensity is zero and variance is one. The first and second convolutions are followed by the average pooling layer. Before the last fully connected layer, we used a dropout of 0.25, which converges the training process faster and increases the accuracy. The output of the last convolution layer is the feature embedding of the ROI under study. These features are further fed to the fully connected layers for binary classification. Adding a dropout of 0.25 in the first fully connected layer improved the accuracy. We used SoftMax as the last layer activation and cross-entropy as the loss function. For faster training and to avoid dying ReLU problem we have utilized Leaky ReLU activation in other layers with *α* = 0.3 [48]. Despite the use of sobolev and other gradient based optimizers in some recent studies [49], we have applied Adam optimizer [50] by considering its fast convergence and efficiency. The Xavier initialization [51] was used for weight and bias initialization. The total number of parameters in the network is 100,197, among which 99,925 parameters are trainable. We tried different structures and hyperparameters. We determined the proposed network after several trials.

### MRI classification

Let C = {aAD, ADD, mAD, NC} be the categories, O = {(ADD, NC), (ADD, mAD), (ADD, aAD), (mAD, NC), (mAD, aAD), (aAD, NC)} be the classification objectives, and R = {LH, RH, LA, RA, LI, RI} be the ROIs. A classifier M_l,i,j_ produces a sequence of scores S(s_l,i_,s_l,j_) for a sequence of TVPs (t_l,1_, t_l,2_, … t_l,n_) generated from R. The scores in favor of C_l,i_ and C_l,j_ for each TVP t_l,i_ are summed up to compute the region-based score of an MRI. The score is SoftMax normalized using Eq 6.

$$\begin{array}{c} S_{l,i}=\frac{\sum_{k=1}^{n}e^{s_{l,i,k}}}{\sum_{k=1}^{n}\left(e^{s_{l,i,k}}+e^{s_{l,j,k}}\right)};\;S_{l,j}=\frac{\sum_{k=1}^{n}e^{s_{l,j,k}}}{\sum_{k=1}^{n}\left(e^{s_{l,i,k}}+e^{s_{l,j,k}}\right)} \end{array}$$ (6)

Here, *s*_*l*,*i*,*k*_ and *s*_*l*,*j*,*k*_ are the scores for *t*_*l*,*k*_ in favor of class *C*_*l*,*i*_ and *C*_*l*,*j*_. *S*_*l*,*i*_, and *S*_*l*,*j*_ are the normalized scores for classes *C*_*l*,*i*_ and *C*_*l*,*j*_. e

**Algorithm 2**: Algorithm for MRI Classification

**Data**: I:a test sMRI scan; S:Scan that has ROI labels

Y: label of I, trained model set, M = m_i,j,l_, location label L = {LH:17, RH:53, LA:18, RA:54, LI:1035, RI:2035}, classification tasks O = {(ADD, NC), (ADD, mAD), (ADD, aAD), (mAD, NC), (mAD, aAD), (aAD, NC)}

**Result**: A table S containing class probability score of C = [c_i_, c_j_]. c_i_ and c_j_ ∈ {aAD:c_1_, ADD:c_2_, mAD:c_3_, NC:c_4_}

1 *Data* ← *testData*

2 *S* ← 0

3 **for** *Obj*(*i*, *j*) ∈ *O* **do**

4  **for** *l* ∈ *R* **do**

5   *x*, *y* ← *Data*[*l*, *i*, *j*]

6   *m* ← *M*[*l*, *i*, *j*]

7   *score* ← *m*(*x*))

8   $S_{l,i}=\frac{\sum_{k=1}^{n}e^{s_{l,i,k}}}{\sum_{k=1}^{n}(e^{s_{l,i,k}}+e^{s_{l,j,k}})}$

9   $S_{l,j}=\frac{\sum_{k=1}^{n}e^{s_{l,j,k}}}{\sum_{k=1}^{n}(e^{s_{l,i,k}}+e^{s_{l,j,k}})}$

10  **end**

11  $S_{i}=\frac{\sum_{l=1}^{6}e^{S_{l,i}}}{\sum_{l=1}^{6}(e^{s_{l,i}}+e^{s_{l,j}})}$

12  $S_{j}=\frac{\sum_{l=1}^{6}e^{s_{l,j}}}{\sum_{l=1}^{6}(e^{s_{l,i}}+e^{s_{l,j}})}$

13 **end**

14 return S

To determine the most appropriate class label for a given sMRI, the results from all ROI-based models are combined. Each ROI-based model produces decision scores of an sMRI that indicates how well the sMRI fits a class. The individual decisions of the relevant sMRIs are combined. Then, we have performed SoftMax normalization on the scores. The most likely value is selected as the final class for an sMRI. The scores for ensemble classification are determined by Eq 7. The details are depicted in Fig 1 and in algorithm 14

$$\begin{array}{c} S_{i}=\frac{\sum_{l=1}^{6}e^{S_{l,i}}}{\sum_{l=1}^{6}\left(e^{s_{l,i}}+e^{s_{l,j}}\right)};\;S_{j}=\frac{\sum_{l=1}^{6}e^{s_{l,j}}}{\sum_{l=1}^{6}\left(e^{s_{l,i}}+e^{s_{l,j}}\right)} \end{array}$$ (7)

## Experimental setup

### Platform

The experiment was performed in the Python 3.7 environment. We used the TensorFlow GPU 1.8 and Keras 2.4. The operating system was Windows 10 installed on an “Intel(R) Xeon (R) CPU E5-1607 v4 @ 3.10 GHz with 32 GB of RAM” machine. The GPU was NVIDIA Quadro M4000. FreeView was used for viewing and navigating through the images. FreeSurfer was used for preprocessing and measurement purposes.

### Data set separation

The sMRI scans provided in the GARD data set are baseline sMRI scans. All available scans are taken into consideration for the experiment. We divided the data set into a training and testing set. For testing, 50% of each class were kept. The remaining sMRIs from all classes were used for training and validation. As we have used TVP generated from the ROI locations, we did not encounter the data scarcity problem for training. Moreover, we applied shearing, rescaling and zooming of the TVPs for data augmentation purposes to avoid the class imbalance problem during training.

### Ground truth preparation

For data generation, we have considered the label of each voxel in the labeled-sMRI of GARD. If the label of a voxel matched the ROI label, then the same voxel location in the sMRI is used to generate a TVP. The label of the sMRI from which the TVP is obtained is considered the label of the TVP. For training the patch-based CNN, we have used all the voxels in an ROI for TVP generation. For testing purposes, we have taken 32 TVPs for each ROI from each sMRI. The voxel locations were selected semirandomly. The only constraint was that the boundary voxels of a ROI are avoided. The details of the ground truth preparation are illustrated in algorithm 1.

### Training and validation

For patch-based classification, we trained different architectures with different hyperparameters. The presented models were trained for 20 epochs with a batch size of 32. We started the training with a learning rate of 0.001. The learning rate was reduced by a tenth if the validation loss stopped declining for three consecutive epochs. The default parameter settings were used for the optimizers, regularizers and constraints. We used 3-fold cross-validation to train the PBCs. All of the other settings are the same as those in [14]. First, we trained the bare model for AD/NC classification. Then, we retrained the model for AD/aAD. The AD/aAD model was retrained for the AD/mAD classification task. This model was retrained to classify mAD vs aAD. Then, we retrained the previous model for diagnosing aAD from NC. The exponential decay rates for the first and second moment estimates are 0.9 and 0.999, respectively.

## Results

We have evaluated 36 different models trained for six different ROIs and six classification tasks. The evaluation outcomes are summarized in Table 2.

**Table 2:** Performance of the trained classifiers.

**Region of Interest**	**ADD vs NC**						**ADD vs mAD**					
	Precision	Recall	F1-score	Accuracy	MCC	AUROC	Precision	Recall	F1-score	Accuracy	MCC	AUROC
**Left amygdala**	72.30	77.04	74.60	78.80	0.57	78.03	86.00	70.49	77.47	68.75	0.48	71.44
**Right amygdala**	75.00	78.68	76.80	80.79	.06	83.13	91.30	68.85	78.50	71.25	0.52	77.31
**Left Hippocampus**	86.15	91.80	88.88	90.72	0.81	90.67	92.59	81.96	86.95	81.25	0.68	83.09
**Right Hippocampus**	75.00	78.68	76.80	80.79	0.73	88.43	91.30	68.85	78.50	71.25	0.64	84.12
**Left Insula**	76.27	73.77	75.00	78.72	0.57	81.13	84.78	63.93	72.89	63.75	0.45	68.94
**Right Insula**	72.58	73.77	73.17	76.59	0.52	76.27	84.44	62.29	71.69	62.50	0.36	62.47
**Ensemble**	90.62	95.08	92.80	94.03	0.88	95.41	93.10	88.52	90.75	86.25	0.77	89.21
**Region of Interest**	**ADD vs aAD**						**mAD vs aAD**					
	Precision	Recall	F1-score	Accuracy	MCC	AUROC	Precision	Recall	F1-score	Accuracy	MCC	AUROC
**Left amygdala**	70.31	73.77	72.00	76.82	0.52	77.47	57.14	59.01	58.06	65.56	0.29	66.02
**Right amygdala**	75.80	77.04	76.42	80.79	0.60	80.67	61.53	65.57	63.49	69.53	0.37	70.60
**Left Hippocampus**	81.53	86.88	84.12	86.75	0.72	88.78	66.66	68.85	67.74	73.50	0.45	72.28
**Right Hippocampus**	75.80	77.04	76.42	80.79	0.69	85.02	61.53	65.57	63.49	69.53	0.39	72.19
**Left Insula**	71.18	68.85	70.00	74.46	0.48	75.12	45.94	55.73	50.37	55.62	0.11	57.25
**Right Insula**	62.12	67.21	64.56	70.19	0.39	69.95	48.52	54.09	51.16	58.27	0.37	58.40
**Ensemble**	85.07	93.44	89.06	90.77	0.81	92.59	67.64	73.01	70.22	74.50	0.48	76.05
**Region of Interest**	mAD vs NC						**aAD vs NC**					
	Precision	Recall	F1-score	Accuracy	MCC	AUROC	Precision	Recall	F1-score	Accuracy	MCC	AUROC
**Left amygdala**	65.21	73.77	69.23	73.50	0.46	73.62	44.00	54.09	48.52	53.64	0.07	56.27
**Right amygdala**	65.07	67.21	66.12	72.18	0.43	73.41	41.02	52.45	46.04	50.33	0.01	53.53
**Left Hippocampus**	70.00	80.32	74.80	78.14	0.56	80.18	45.33	55.73	50.00	54.96	0.09	59.55
**Right Hippocampus**	65.07	67.21	66.12	72.18	0.56	82.19	41.02	52.45	46.04	50.33	0.07	57.40
**Left Insula**	54.05	65.57	59.25	63.57	0.27	68.63	41.09	49.18	44.77	50.99	0.01	51.13
Right Insula	52.70	63.93	57.77	62.25	0.25	64.04	40.25	50.81	44.92	49.66	-0.29	50.35
Ensemble	72.97	88.52	80.00	82.11	0.65	82.04	46.66	57.37	51.47	56.29	0.13	60.20

Reported on the GARD dataset.

To evaluate the models, we have taken $\text{accuracy}=\frac{\left(TP+TN\right)}{\left(TP+TN+FP+FN\right)}$, precision or $\text{positive}\;\text{predictive}\;\text{value}\;(\text{PPV})=\frac{TP}{\left(TP+FP\right)}$, specificity or $\text{true}\;\text{negative}\;\text{rate}=\frac{TN}{TN+FP}$, hit rate or sensitivity or recall or $\text{true}\;\text{positive}\;\text{rate}=\frac{TP}{\left(TP+FN\right)}$ and $\text{F1-score}=\frac{\left(2\times precision\times recall\right)}{\left(precision+recall\right)}$ into consideration. Here, TP, TN, FP and FN are acronyms for the number of model-predicted true positive, true negative, false positive and false negative samples, respectively. In addition to the abovementioned metrics, we have evaluated our models with the Matthews correlation coefficient (MCC) to produce a more informative and truthful score and to avoid overly optimistic outcomes [52]. The MCC is defined by $\frac{TP\times TN-FP\times FN}{\sqrt{\left(TP+FP)(TP+FN)(TN+FP)(TN+FN\right)}}$. We have also considered the area under the receiver operating characteristic curve (AUROC) to analyze the performance of the models.

To evaluate each model, we used individual sMRIs as a sample. We generated at least 32 TVPs from each ROI for each test sMRI to obtain its label. First, we fed TVPs to the patch-based classifiers to obtain the decision scores for each individual TVP. We then added the scores of all patches and normalized the scores to obtain decisions based on a single ROI. The single ROI decisions from six different models were further summed up and SoftMax-normalized for the final decision.

### Left hippocampal region-based classifiers

**Fig 2:**
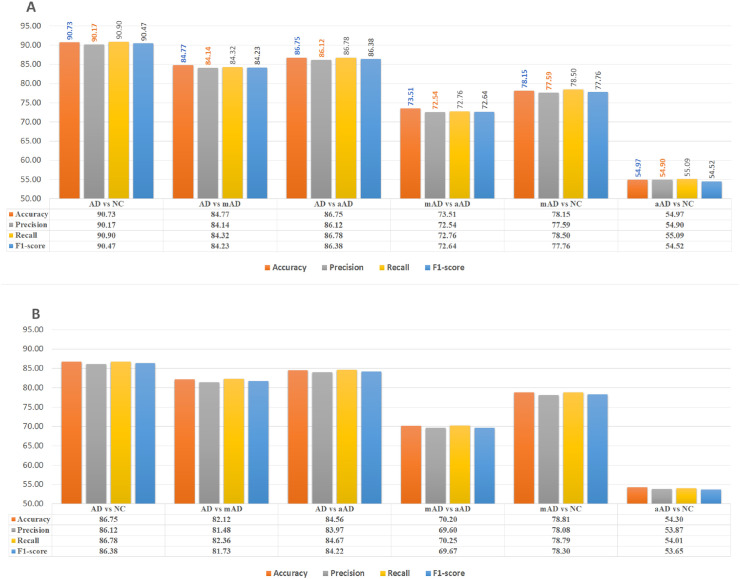
AD/NC, AD/mAD, AD/aAD, mAD/aAD, mAD/NC and aAD/NC classification performance based on test Three-View Patches (TVPs) generated from the hippocampal regions. (A) Performance of CNN classifiers based on TVPs generated from the left hippocampus (LH). (B) Performance of CNN classifiers based on TVPs generated from the right hippocampus (RH).

Fig 2A demonstrates the classification performance of all pairs of classes based on left hippocampal features.

We have observed 90.73% accuracy in classifying ADD over NC. The precision and recall for this task are 90.17% and 90.90%, respectively. The F1-score performance was 90.47%. The MCC was 0.81. The AUROC was observed to be 90.67%. The false discovery rate of this model was 10%.

The classification accuracy of ADD subjects from mAD is 81.25%, with precision, recall and F1-score values of 92.59%, 81.96% and 86.95%, respectively. The AUROC and MCC for this model were computed as 83.09% and 0.55, respectively.

Classifying the mAD scan from NC scans showed a TPR of 80.32%, with a false discovery rate of 23.33%. The accuracy was 78.14%, with MCC = 0.56. The F1-score and PPV values for this classification were 74.80% and 70%, respectively. The AUROC was 80.18%.

The mAD/aAD classification accuracy was 73.51%, with a true positive discovery of 68.85% and a false detection rate of 23.33%. The PPV and F1-score values were 66.67% and 67.74%, respectively. The AUROC was 72.28%, with MCC = 0.45.

The diagnostic accuracy (86.75%) for ADD scans from the aAD scan is better than that for the ADD/mAD classification tasks. We have also observed improved true positive rates (by at least 5%) and reduced false detection rates (by almost 8%). The PPV for this model was 81.54%, while the F1-score and MCC was 84.21% and 0.72, respectively. A better AUROC (88.78%) was observed as well.

Classifying aAD scans from NC scans showed limited performance, with MCC = 0.09, which is close to zero. The accuracy was 54.96%, with AUROC = 59.55%. The false alarm rate was 45.46%, and true detection rate was 55.73%. The F1-score was 50%, and the PPV was 45.33%.

### Right hippocampus region-based classifiers

The classification performance of all pairs of classes based on right hippocampus features is demonstrated in Fig 2B. The right hippocampus model accurately differentiated 86.75% of the ADD MRIs from their NC counterparts. Approximately 86.12% of ADD scans were correctly diagnosed as ADD, and a total of 86.67% of cases of ADD diagnosed by MRIs were true ADD. The PPV and F1-score values were 41.54% and 84.13%, respectively. The AUROC was 88.43%, with MCC = 0.73. The right hippocampus provides useful information to classify ADD vs mAD, with an accuracy of 82.5%. The PPV and F1-score for this task were 92.72% and 87.93%, respectively. The detection rate was 83.61%, while the false discovery was approximately 20%. The MCC and AUROC were 0.57 and 84.12%, respectively. The features from this region classified mAD scans from NC MRIs with nearly 78.81% accuracy. The true positive rate was 78.69%, while the false detection rate was 21.11%. The F1-score and PPV were 75% and 71.64%, respectively. The AUROC was 82.19%. The MCC of the model was 0.73.

The ADD vs aAD classification performance was observed to be 84.56%, with a true detection rate of 85.25% and a false diagnosis rate of approximately 16%. The PPV and F1-score were 78.78% and 81.89%, respectively. The AUROC was 85.02, while the MCC was 0.69.

Right hippocampus features differentiated 70.20% of mAD scans from aAD scans, with a PPV of 61.43% and a true detection rate of 70.49%. The false diagnosis rate was 30%. The F1-score was 65.65%. The MCC and AUROC of the model were 0.40 and 72.19%, respectively.

The classification performance for distinguishing between aAD and NC is 54.30%, with MCC = 0.079 and AUROC = 57.4. The false diagnosis is approximately 45%, with PPV = 44.44%. The disease discovery rate is 52.46%, with F1-score = 48.12%.

### Left amygdala region-based classifiers

**Fig 3:**
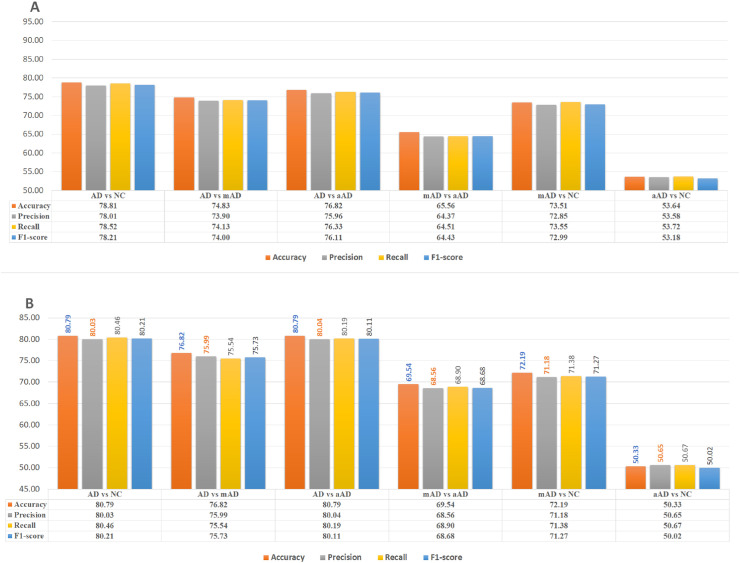
AD/NC, AD/mAD, AD/aAD, mAD/aAD, mAD/NC and aAD/NC classification performance based on test Three-View Patches (TVPs) generated from amygdala regions. (A)Performance of CNN classifiers based on TVPs generated from the left amygdala (LA). (B) Performance of CNN classifiers based on TVPs generated from the right amygdala (RA).

The left amygdala features were also significant in ADD diagnosis. The accuracy was 78.81%, with a significant MCC (0.57) for the ADD/NC classification task. The left amygdala-based AD/NC classification model correctly recognized 72.30% of ADD MRIs, while 77.05% of ADD diagnosed MRIs were actually ADD. The PPV and F1-score were 72.30% and 74.63%, respectively. The false detection rate was 20%. The AUROC was 78.03%. The left amygdala was also observed to provide discerning features for diagnosing the mAD stage. The features from this ROI provided 68.75%, 65.56% and 73.51% accuracy for detecting mAD from the ADD, aAD and NC MRIs, respectively, with significant MCC values (0.57, 0.29 and 0.46, respectively); the AUROC values were 71.44%, 72.28%, and 73.62%. The false detection rates for these tasks were 37%, 30% and 26%, respectively, while the true diagnosis rates were 70.50%, 59.02% and 73.77%. The PPV and F1-score values for the tasks were 86%, 57.14% and 65.22% and 77.48%, 58.06% and 69.23%, respectively. Moreover, 53.64% of aAD MRIs were diagnosed correctly from the aAD vs NC classification experiment using this ROI feature. The MCC value is close to zero, and the false detection rate is approximately 47%. Fig 3A demonstrates the classification performance of all pairs of classes based on left amygdala features.

### Right amygdala region-based classifiers

The right amygdala provides distinctive features for diagnosing ADD MRIs from NC, mAD and aAD scans, with 80.79%, 71.25% and 80.79% diagnostic accuracy, respectively, with MCC = 0.6, 0.41 and 0.6, respectively; the PPV and F1-score values were 75.00%, 91.30% and 75.80% and 76.80%, 78.50% and 76.40%, respectively. The false discovery rates were 17.88%, 21% and 16%, respectively; the true detection rates were 78.68%, 68.85% and 77.05%; and the AUROC values were 83.13%, 77.31% and 80.67%.

From the features of this region, 69.54% and 72.18% of MRIs were observed to be correctly classified in mAD/aAD and mAD/NC classification tasks, respectively. Here, the right amygdala provided limited features for diagnosing aAD MRIs from NC MRIs (only 50.33% binary classification accuracy). Fig 3A demonstrates the classification performance of all pairs of classes based on right amygdala features.

### Left insula region-based classifiers

The left insula were observed to provide significant and distinctive features for classifying ADD MRIs over NC, with an accuracy of 78.72%, which is nearly equivalent to that for the left amygdala (%), with MCC = 0.57. The PPV and TPR values were 78.38% and 78.14%, respectively. The F1-score and AUROC were 78.24% and 81.13%, respectively.

This ROI demonstrated 63.75% accuracy for classifying ADD vs mAD, with an MCC of 0.68. The AUROC and PPV values were 68.94% and 84.77%, respectively. The false diagnostic rate for mAD was approximately 15%. The F1-score and TPR values were 90.47% and 90.90%, respectively.

Moreover, 74.47% accuracy was observed for diagnosing ADD from aAD scans, while the diagnostic accuracy for aAD from NC was 54.97%. The MCC values for these tasks were 0.47 and 0.01, respectively, with false diagnostic rates of 13% and 45%. The TPRs were 86.78% and 55.09%, respectively, while the PPVs were 86.12% and 54.90%.

The diagnosis rate for mAD from aAD and NC was 73.51% and 63.58%, respectively, while the MCCs were 0.11 and 0.27 for the same tasks.

**Fig 4:**
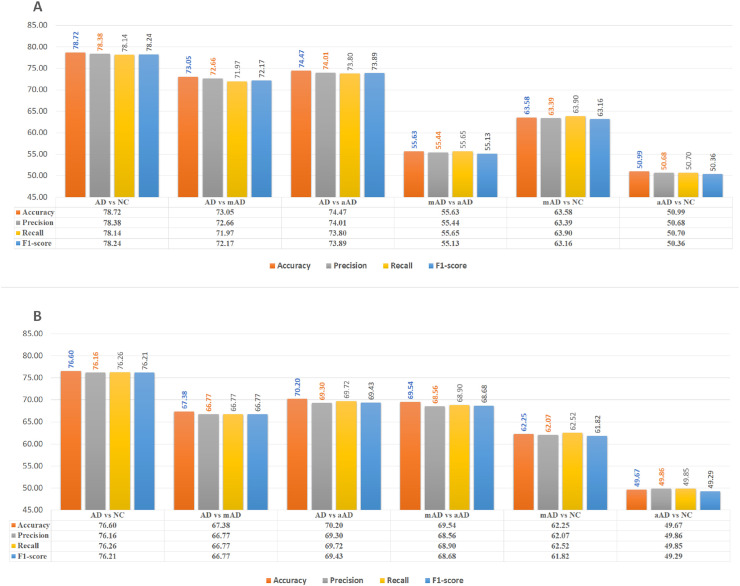
AD/NC, AD/mAD, AD/aAD, mAD/aAD, mAD/NC and aAD/NC classification performance based on testing Three-View Patches (TVPa) generated from insula regions. (A) Performance of CNN classifiers based on TVPs generated from the left insula (LI). (B) Performance of CNN classifiers based on TVPs generated from the right insula (RI).

The left insula was observed to show no distinctive features for classifying aAD and NC (50.90%), with an MCC of 0.01. The TPR and false detection rates were almost 50.00%. Fig 4A demonstrates the classification performance of all pairs of classes based on left insula features.

### Right insula region-based classifiers

Based on right insula features, the corresponding model accurately diagnosed 76.16% of ADD MRIs as ADD, and a total of 76.26% of ADD-diagnosed MRIs were actually ADD labeled MRI, while the overall diagnostic accuracy based on the right insula feature was 76.60%. The MCC and AUROC values were 0.72 and 76.27%, respectively.

The classification accuracy of ADD scans from mAD and aAD sMRIs were 62.5% and 70.2%, respectively. The right insula features accurately classified 62.25% of mAD scans from their NC counterparts, while the accuracy for mAD vs aAD was observed to be 58.28%. However, the aAD sMRIs classification from NC is nearly random (50.33%). Fig 4B demonstrates the classification performance of all pairs of classes based on right insula features.

### Results of ensembles

**Fig 5:**
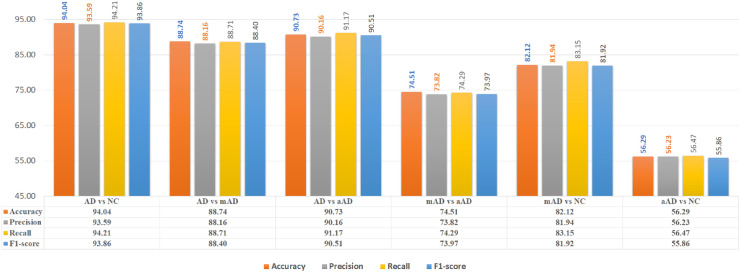
AD/NC, AD/mAD, AD/aAD, mAD/aAD, mAD/NC and aAD/NC classification performance based on six selected regions (hippocampi, amygdalae, insulae).

The ensemble of the six models is shown in Fig 5. The overall accuracy for ADD diagnosis from NC of the ensemble model was 94.03%, with AUROC = 85.41% and MCC = 0.88. The precision for the ADD class was 93.63%, while it was 96.552% for the NC class. In addition, the recall scores were 95.08% and 93.33% for the ADD and NC class, respectively. The F1-scores for the ADD and NC class were 92.8% and 94.91%, respectively.

The overall accuracy for ADD diagnosis over mAD scans was 86.25%, with AUROC = 89.11% and MCC = 0.77. The false detection rate and true positive rate were 12% and 88.71%, respectively. The classifier demonstrated an F1-score of 93.86% and a PPV of 88.74%.

ADD diagnosis over aAD showed little improvement, with accuracy = 90.73%, AUROC = 92.59%, MCC = 0.81, PPV = 90.16%, TPR = 90.16% and F1-score = 90.51%.

The mAD diagnosis from aAD and NC demonstrated an accuracy = 74.51% and 81.94%, respectively, MCC = 0.48 and 0.65, and AUROC = 76.05% and 82.04%. The false detection rates were 26% and 18%, respectively, while the TPR values were 74% and 83% and the PPVs were 73% and 82%.

## Discussion

In this study, we proposed a deep learning framework for staging AD based on the selected ROIs of the sMRI modality. We used the permutation test on volumetric measurement of the GARD cohort data set to find the most affected regions. CNN classifiers were trained based on the TVPs from those selected regions of sMRI scans. To the best of our knowledge, the aAD stage was not considered for diagnosis in previous studies. Here, we have also considered the aAD stage of AD. Our study has an important contribution for clinical practice in assessing the symptoms of patients and providing the earliest diagnosis of AD. In our study, when applying the CNN to learn features from ROI-TVPs, a more detailed representation is provided, and therefore, significant improvements have been achieved by the proposed methods. After stacking the ROI-TVP models, a higher-level representation is obtained. Therefore, the ROI-based ensemble CNN achieves performance comparable to that of the state-of-the-art methods.

This work demonstrated the significance of the hippocampi, amygdalae and insulae for staging the AD spectrum. Each of the mentioned ROIs are individually analyzed with the proposed TVPCNN. Ensembles of TVPCNN were deployed to analyze the combined contribution of all ROIs.

### Significant regions and landmarks

Here, from the volumetric analysis of the GARD data set, we found that the hippocampus, amygdala, insula, parahippocampus, precuneus, enthorhinal cortex, gray matter, and CT were AD-affected brain regions. These regions are explainable with AD pathology. The existing literature also supports the results of the permutation test. For example, the hippocampus region is the earliest to be severely affected by AD [53–55].

The amygdala in the temporal lobe is essential for memory, and damage in this region by AD can explain memory loss [16]. Pathologic changes within the insula may be responsible for the behavioral dyscontrol and visceral dysfunction that often occur in AD [56–58].

**Fig 6:**
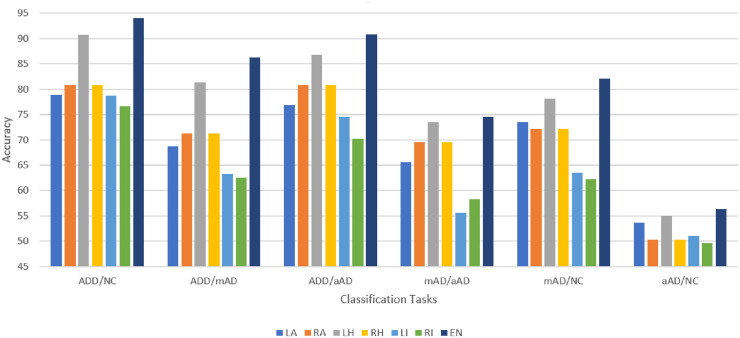
AD/NC, AD/mAD, AD/aAD, mAD/aAD, mAD/NC and aAD/NC classification accuracy based on test Three-View Patches (TVPs) generated from the ROIs. Reported on GARD data.

**Fig 7:**
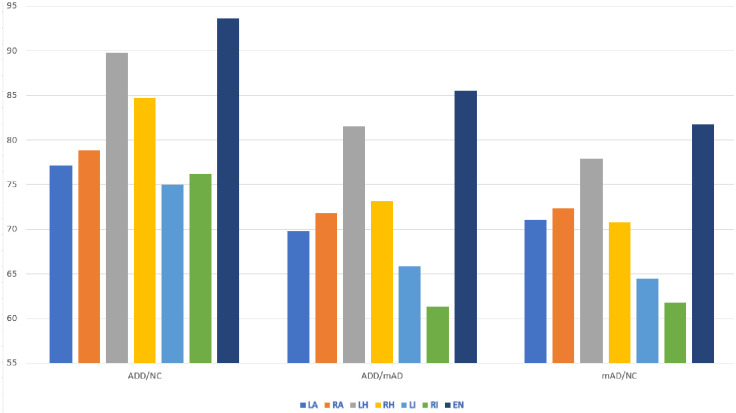
AD/NC, AD/mAD,and mAD/NC classification accuracy based on testing Three-View Patches (TVP) generated from the regions of interests. Reported on ADNI data.

Another observation is that the CNN supports the permutation test outcomes. We found similarity between the identified disease-related regions from the statistical test and CNN models, see Figs 6 and 7. In the permutation test, the hippocampi were observed to be the most affected regions. The specificity and sensitivity analyses of the CNN classifiers also confirm that the hippocampi provide the most discerning features for AD staging. The features provided by the amygdalae and insulae are also significant for clinical decision making.

### Patch-based CNN classifiers

The study was performed without utilizing the whole brain. We have generated TVPs of selected ROIs to test the performance of the trained models. We have deployed CNN models for classification despite lacking in incorporating spatial information. We did not consider the recent update such as Capsule network in order to address our primary issue of the experiment which is to find the significance of the selected ROIs for AD spectrum analysis. In future we may conduct an experiment in this regard.

Our TVPCNN approach provides us with multiple prospective benefits [14]. Moreover, utilization of dropout [59] and batch normalization [60] ensures better generalization. The CNN for ADD/NC classification was trained first. To fix the hyperparameters and constraints of the network, we have employed a trial-and-error approach, starting with LeNet-5 along with SoftMax as the classifier. After training and evaluating the ADD/NC classifier, an instance of this model was retrained for mAD/NC classification. We transferred the knowledge of one classification task to another in the following order: ADD/NC, mAD/NC, ADD/aAD, ADD/mAD, mAD/aAD, and aAD/NC. We kept the data distribution of the successor model the same as the input distribution of the predecessor classifier. The parameters, structure, constraints and regularizers were also kept the same. For each ROI, the process was repeated. The training and validation was performed on TVP data, while the testing was done in a scan-wise manner.

To determine the label of an MRI based on individual TVP decisions, we have considered two alternative approaches, namely, maximum count and score aggregation. In the maximum count approach, we have considered each TVP-based decision as a vote in favor of a class label. The class label is determined based on maximum votes. Each TVP decision has an equal weight for determining the class label. In the score aggregation approach, the TVP-based decisions are added and then SoftMax normalized. Next, the class label is determined from the SoftMax score. In this approach, each TVP-based decision has a weighted contribution in determining the class label of an MRI. Our observations confirmed that the score aggregation approach outperforms the maximum count approach. The reason behind this finding may be the strong evidence (higher score) that the minority patches contributed more than the poor support of the majority patches in determining the class label of an MRI.

### Comparison with existing models

The existing deep learning-based studies for early diagnosis of AD may be broadly categorized into 1) patch-based, 2) region-based, 3) slice-based, and 4) voxel-based approaches. In patch-based studies, 3D patches are taken into consideration. In region-based studies [11, 61, 63, 65, 66], the specific region of interest information is used. Slice-based studies [12, 64] take the axial, sagittal or coronal slices for diagnosis. Voxel-based studies [22, 23, 67] consider voxel intensities for the whole brain or tissue components. In Table 3, we have summarized the methods with findings.

**Table 3:** Comparison of the proposed approach with state-of-the-art approaches.

Ref	Dataset	Modality	Model Feed	Method	Result		
					AD/NC	AD/mAD	mAD/NC
[12]	ADNI	MRI+PET	2D Slice	MMSDPN+ LKSVM	96.93 ± 4.53	86.99+-4.82	87.24 ± 4.52
[7]	ADNI	MRI+PET	Voxel+3D Patch	MMDBM+SVM	92.38 ± 5.32	75.92 ± 15.37	84.24 ± 6.26
[61]	ADNI	MRI+PET+CSF	Region	Stacked AE+ MKSVM	0.89 ± 0.014	0.689 ± 0.023	0.737 ± 0.025
[61]	ADNI	MRI+PET+CSF+Clinical	Region	Stacked AE+ Sparsed AE+ MKSVM	0.899 ± 0.014	0.689 ± 0.023	0.737 ± 0.025
[22]	ADNI	MRI	Voxel	Sparse AE + 3DCNN	95.39%	86.84%	92.11%
[11]	ADNI	MRI+PET	Region	Stacked Sparse AE+Zero Mask+ SoftMax	91.40 ± 5.56	-	82.10 ± 4.91
[62]	ADNI +MIRIAD	MRI	3D Patch + ROI	3DCNN	91.09	-	-
[63]	ADNI	MRI+PET	Region	Ensemble DBN+SVM	0.90 ± 0.08	0.84 ± 0.09	0.83 ± 0.14
[64]	OASIS+ Local Data	MRI	Slice	2D CNN	97.65	-	-
[65]	ADNI	MRI	Region	Sparse Regression + 2DCNN	91.02	69.19 ± 8.19	-
[66]	ADNI	MRI+PET+CSF	Region	PCA RBM SVM	91.4 (1.8)	77.4 (1.7)	70.1 (2.3)
[23]	CADDementia+ ADNI	MRI	Voxel	3D-ACNN	97.6+-0.6	95+-1.8	90.8+-1.1
[67]	ADNI	MRI	Voxel	RESNET	80 ± 07	63 ± 09	61 ± 10
Proposed Approach	GARD	MRI	2D Patch+ ROI	2DCNN	94.04	86.25	82.12
	ADNI	MRI	2D Patch+ ROI	2DCNN	93.58	85.51	81.73

Our method utilizes the benefits of slice-, patch- and region-based methods in a single modality. We have taken patches from axial, sagittal and coronal slices from statistically significant brain regions. The proposed method demonstrates comparable accuracy even though we have used a lightweight CNN. [12, 22, 23, 64] demonstrated better performance in all three classification tasks because these methods used multimodal data or whole brain information along with a complex model deployment.

To compare with the state-of-the-art methods we have retrained and tested the models on ADNI data. The results are presented in Fig 7 and Table 4. Our experiments demonstrated that 2D patch-based training of a deep CNN may provide the expected outcome in terms of diagnosis and efficiency. Our approach also demonstrated that a simple and efficient CNN can be designed using sMRI data as an efficient CAD system. We used only small patches of size 32 × 32 from the selected ROIs of the brain sMRIs and achieved comparable accuracy. Hippocampi, amygdalae and insulae provide approximately similar diagnosis results to those of state-of-the-art methods.

**Table 4:** Performance of the trained classifiers.

**Region of Interest**	**ADD vs NC**				
	Precision	Recall	F1-score	Accuracy	MCC
**Left amygdala**	75.72	75.92	75.82	77.17	0.52
**Right amygdala**	77.69	77.21	77.43	78.86	0.54
**Left Hippocampus**	89.74	89.74	89.74	89.74	0.80
**Right Hippocampus**	83.94	84.19	84.06	84.68	0.68
**Left Insula**	74.17	74.45	74.29	75	48.61
**Right Insula**	75.89	76.18	75.97	76.19	0.52
**Ensemble**	92.78	93.88	93.25	93.58	0.87
**Region of Interest**	**ADD vs mAD**				
	Precision	Recall	F1-score	Accuracy	MCC
**Left amygdala**	69.61	69.80	69.62	69.77	0.39
**Right amygdala**	71.81	71.81	71.81	71.81	0.44
**Left Hippocampus**	81.28	81.51	81.36	81.51	0.63
**Right Hippocampus**	73.13	73.05	73.06	73.10	0.46
**Left Insula**	65.74	65.62	65.64	65.83	0.32
**Right Insula**	61.25	61.31	61.23	61.32	0.23
**Ensemble**	85.37	85.52	85.43	85.51	0.71
**Region of Interest**	**mAD vs NC**				
	Precision	Recall	F1-score	Accuracy	MCC
**Left amygdala**	69.46	69.61	69.53	71.09	00.40
**Right amygdala**	71.03	71.19	71.10	72.32	0.43
**Left Hippocampus**	76.64	76.88	76.75	77.88	0.54
**Right Hippocampus**	69.64	69.87	69.74	70.77	0.4
**Left Insula**	64.32	64.20	64.22	64.46	0.29
**Right Insula**	61.69	61.73	61.67	61.76	0.24
**Ensemble**	80.53	81.79	80.59	81.73	0.63

Reported on the ADNI dataset.

Our patch generation reduces the scarcity of training data for generalization. Using the ensemble technique also contributed to building a robust model while avoiding the overfitting problem. Moreover, this approach has helped to avoid obtaining an over-capacity network regarding the training time.

Though, Ensembles of TVPCNN is the first to analyze NIA-AA defined AD spectrum, the method did not demonstrate better classification accuracy for aAD MRIs over NC MRIs. The whole brain computation and multi-modal analysis of the same ROIs would also increase the performance of other classification tasks though considering TVPs from selected ROIs are providing comparable performance.

## Conclusion

In this paper, we have exploited TVP-based CNN classifiers to stage AD with the sMRI modality. The GARD sMRI data set was employed in the experiment. We have considered all class labels as suggested by NIA-AA (aAD, mAD, ADD, and NC). The study confirmed that the hippocampi, amygdalae and insulae provided distinctive features for the diagnosis of ADD and mAD. The true positive diagnostic rates of the learned models were 95.08% (AD/NC), 88.52% (AD/mAD), 93.44% (AD/aAD), 73.02% (mAD/aAD), 88.52% (mAD/NC) and 57.38% (aAD/NC), while the false positive rates were 9.38%, 15.63%, 14.93%, 32.35%, 27.03% and 53.33%, respectively. The highest false positive rate and lowest true positive rate in diagnosing aAD imply that our ROI-based models do not provide sufficient information for the diagnosis of aAD from the sMRI modality. Our findings confirm that simple and efficient methods can be deployed as a CAD system without compromising the performance to assist the physician’s diagnosis. The replication of the experiment with ADNI data also verifies our findings.
